# Implementing patient-centred cancer care: using experience-based co-design to improve patient experience in breast and lung cancer services

**DOI:** 10.1007/s00520-012-1470-3

**Published:** 2012-04-29

**Authors:** Vicki Tsianakas, Glenn Robert, Jill Maben, Alison Richardson, Catherine Dale, Theresa Wiseman

**Affiliations:** 1 Florence Nightingale School of Nursing and Midwifery, King’s College London, 57 Waterloo Road, London, SE1 8WA UK; 2 National Nursing Research Unit, Florence Nightingale School of Nursing and Midwifery, King’s College London, 57 Waterloo Road, London, SE1 8WA UK; 3 School of Health Sciences, University of Southampton & Southampton University Hospitals Trust, Highfield, Southampton SO17 1BJ UK; 4 Integrated Cancer Centre, King’s Health Partners, London, UK; 5 Nursing, Rehab and Quality, The Royal Marsden NHS Foundation Trust, London, UK

**Keywords:** Experience-based co-design, Quality improvement, Breast cancer, Lung cancer, Patient experience

## Abstract

**Purpose:**

The aim of this paper was to briefly describe how the experience-based co-design (EBCD) approach was used to identify and implement improvements in the experiences of breast and lung cancer patients before (1) comparing the issues identified as shaping patient experiences in the different tumour groups and (2) exploring participants' reflections on the value and key characteristics of this approach to improving patient experiences.

**Methods:**

Fieldwork involved 36 filmed narrative patient interviews, 219 h of ethnographic observation, 63 staff interviews and a facilitated co-design change process involving patient and staff interviewees over a 12-month period. Four of the staff and five patients were interviewed about their views on the value of the approach and its key characteristics. The project setting was a large, inner-city cancer centre in England.

**Results:**

Patients from both tumour groups generally identified similar issues (or 'touchpoints') that shaped their experience of care, although breast cancer patients identified a need for better information about side effects of treatment and end of treatment whereas lung cancer patients expressed a need for more information post-surgery. Although the issues were broadly similar, the particular improvement priorities patients and staff chose to work on together were tumour specific. Interviewees highlighted four characteristics of the EBCD approach as being key to its successful implementation: patient involvement, patient responsibility and empowerment, a sense of community, and a close connection between their experiences and the subsequent improvement priorities.

**Conclusion:**

EBCD positions patients as active partners with staff in quality improvement. Breast and lung cancer patients identified similar touchpoints in their experiences, but these were translated into different improvement priorities for each tumour type. This is an important consideration when developing patient-centred cancer services across different tumour types.

## Introduction

Policy-makers increasingly believe that encouraging patients to play a more active role in their healthcare could improve quality, efficiency and health outcomes[1]. Healthcare providers and health systems are trying to make the care they provide more ‘patient-centred’[2] by ensuring that it is delivered in a way that fulfils patients’ needs. The Picker Commonwealth Programme (1988) described patient-centred care (PCC) as that which places patient experience at the centre of service development and provision[3]; patient-centred cancer care is delivered by providing ‘the best outcomes, service and value in health care to every patient, every day.’[4]

To help guide quality improvement efforts, local and national patient survey data are available in some countries, but there is scepticism about their validity [5]; such data do not always indicate how patients’ experiences can be improved and patients often suggest that their experiences cannot be fully captured by pre-determined, ‘tick box’ survey questions. Stacey and colleagues[6] stress the importance of including users’ emotions when designing service systems—not least because resultant improvements in patients’ emotional wellbeing can improve recovery rates—but this requires more detailed qualitative data collection[7]. Whatever the data collection methods, a co-ordinated strategy is then required to use patient feedback to improve services. Several studies have reported improvements following systematic gathering of patient feedback in hospitals[4, 8–11]. However, such an approach is not a priority for many cancer services primarily because organisations lack adequate systems for co-ordinating data collection, assessing its quality and acting systematically on the results[12].

Goodrich and Cornwell[13] describe a number of promising interventions and methods at three levels of an organisation (individual, micro-system, institutional), but there are few evaluations of these. This paper reports one such intervention at the clinical micro-system level that sought to design better experiences for both staff and patients by adopting a user-centred orientation within a participatory, collaborative change process[14]. Drawing on concepts from the design sciences and professions, experience-based co-design (EBCD) focuses on how staff and patients move through and interact with different parts of a service[8]. Patients and staff share their respective experiences, identify and agree improvement priorities and work together to achieve them. EBCD has been applied successfully in a UK National Health Service head and neck cancer service [15] and emergency services in Australia[11, 16]. An independent evaluation of the latter found that this approach succeeded in engaging consumers in ‘deliberative’ processes that were qualitatively different from conventional consultation and feedback techniques and had resulted in wider, and sustained, impacts than those originally envisaged.

This project used the EBCD approach to enhance experiences for breast and lung cancer service patients. In this paper, we briefly describe the process by which patients and staff in these two services identified and implemented improvements in patient experience before (1) comparing the issues identified as shaping patient experiences in the different tumour groups and (2) exploring participants’ reflections on the value and key characteristics of this approach to improving patient experiences.

## Methods

### EBCD approach

EBCD is a form of participatory action research[17] that seeks to capture and understand how people actually experience a process or service. EBCD improves users’ experience by deliberately drawing out the subjective, personal feelings of service users, carers and staff to identify touchpoints—key moments that shape a person’s overall experience[18].

**Fig. 1:**
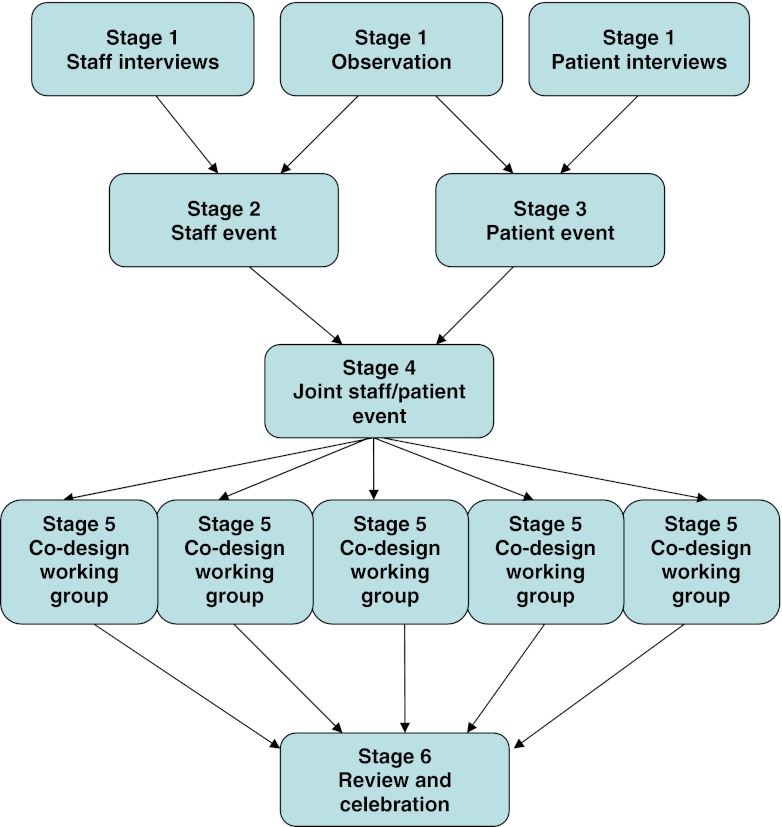
The experience-based co-design (EBCD) process

Figure 1 depicts the EBCD process which began with a 4-month data collection period (stage 1). Thirty six (23 breast, 13 lung) patients were recruited through clinical nurse specialists (CNSs) who were asked to ensure inclusion of patients with a variety of backgrounds, socio-economic status, age groups and experience. An experienced qualitative researcher (VT) conducted filmed, narrative, unstructured interviews lasting 1 to 3 h in which patients described their experiences of care since first diagnosis. Each patient was sent their own film to view before deciding whether it could be shared with other patients and staff. Two researchers (VT and TW) viewed the films independently to ensure analytical rigour and shared understanding of significant touchpoints. They analysed the films by identifying themes (or touchpoints) that shaped overall patient experiences. Films were then edited to produce one composite 35-min film for each service, representing all the key touchpoints. In addition, audio recordings of the narrative interviews were transcribed verbatim and the data analysed thematically (VT and JM) for each tumour group.

A wide variety of staff (from receptionists to lead clinicians) were also interviewed about their experience of working within these services. The 63 (37 breast, 26 lung) staff interviews were transcribed and analysed thematically by three researchers (VT, TW, JM). Participant observation helps to contextualise and understand the patient pathway from patient and staff perspectives[19]. Within the EBCD process, two researchers (VT and TW) conducted a total of 219 h of participant observation of the clinical areas along the patient pathway. Specific aspects of care to be observed were not pre-determined but the observation was intended to focus on both functional and relational aspects[20]. Practice observation and staff interviews were integral to the EBCD approach, but these data are beyond the scope of this paper. An independent process evaluation of the whole project is available elsewhere[21].

Following the data collection, staff met to review the themes arising from the staff interviews and observational data in order to identify their priorities for improving services (stage 2). Stage 3 saw patients invited to a showing of an edited 35-min film after which a facilitated group exercise enabled discussion of emerging issues. An emotional mapping exercise was used which involved patients reflecting together on the emotional impact of the touch points along the pathway[22]. Following this group work, patients voted on their shared priorities for improving services, based on these group reflections.

Staff and patient priorities were presented at a joint event (stage 4) at which staff viewed the patient film for the first time. Mixed groups of patients and staff used the issues highlighted in the film together with the priorities from the separate staff and patient meetings as a basis to identify joint priorities for improving services. Based on these joint priorities, patients and a variety of medical, allied health professional and administrative staff volunteered to join specific ‘co-design working groups’ to design and implement improvements to services (stage 5). The majority of these groups were facilitated by service improvement leads and ground rules were established from the outset, ensuring all participants had equal voices. At stage 6, these separate co-design working groups reconvened to discuss their work to date and plan the next stages of the improvement process.

### Setting

The EBCD process was implemented in breast cancer and lung cancer services within a comprehensive cancer centre in England in 2009–2010. The centre aspired ‘to deliver internationally distinctive cancer services, forming a fully Integrated Cancer Centre that is amongst the top 10 globally and where the care that is provided is patient centred, research driven and clinically led’. Breast services were selected due to the ambulatory nature of the pathway and the high number of patients. Lung services were selected due to the complex nature of presentation and pathway. There was a small budget to make service changes, but for the most part, service changes were cost neutral. For some changes, such as the introduction of the welcome DVD, funds were requested from charity sources. Trust service managers were engaged with the project from the outset and endorsed service development and change.

### Data analysis for this paper

Two researchers (VT and TW) compared and contrasted the touchpoints that were identified in each of the breast and lung qualitative data sets. Findings for each of the tumour groups were tabulated in order to compare touchpoints, improvement priorities and improvement outcomes.

## Results

The key touchpoints, improvement priorities and ‘outcomes’ for each service are described below.

Breast and lung cancer patients identified similar touchpoints at particular moments along the patient pathway. Tables 1 and 2 illustrate how these touchpoints translated into tumour-specific improvement priorities and the subsequent improvement ‘outcomes’. Both patient groups reported very positive experiences in radiotherapy and chemotherapy which did not result in improvement priorities or co-design groups and are therefore omitted from the findings.

**Table 1:** Breast co-design working groups and outcomes

Working group	Outcomes of co-design group work
Day surgery	• Patients no longer separated from loved ones early in the process
	• Establishment of dedicated consultation room—offers more privacy and dignity
	• Lead for breast surgery reviewed information flow from pre-assessment to post-surgery
	• Physiotherapists identified best time to offer patients information about exercise
Appointments	• New, efficient appointment processes
	• All newly diagnosed patients agree the date of surgery and subsequent appointments on the day of their results
Communication	Information along the way
	• Much of the breast patient information reviewed and updated
	Better people skills
	• All administrative staff receive customer-care training and are shown patients’ DVD
	• Healthcare assistants’ interpersonal skills assessed prior to recruitment
	• Managers and administrative staff use values-based performance tool which can improve patients’ experience
	Clinic-related issues
	• Changes to structure of clinics to reduce waiting times
	• Patients regularly updated about waiting times in clinic
	• All staff names displayed on noticeboard
	• Designated phlebotomist has reduced waiting times for blood tests
Information about symptoms	• Patients receiving same chemotherapy treatment given option to receive information in a group
	• Enhanced processes for accessing support around hair loss

**Table 2:** Lung co-design working groups and outcomes

Working group	Outcomes of co-design group work
Diagnosis	• Establishment of second breaking-bad-news room
	• Guidance on diagnosis procedures included in junior doctors’ induction
	• Improved links between patients and CNSs
Information	• Patients waiting in oncology outpatients encouraged to visit information office
	• Promotion of information and support centres at different sites (advertising at hospital entrance)
	• Patient information leaflets for specific points in the pathway
	• Patient DVD ‘welcome to cancer services’ for newly diagnosed and referred patients
Continuity of care	• Link nurse scheme to improve cross-site working
	• Quarterly CNS forum to facilitate development of service
	• Staff name board (with pictures) enables patients to identify staff members easily
	• Cross-site visibility of test results, email and remote access for staff (IT systems)
	• Improved access to out-of-hours oncology services
Other improvement work (in addition to co-design groups)	• Re-profiling of outpatient clinic booking to reduce waiting times and facilitate patient access to same doctor
	• Establishment of nurse-led end of treatment clinics
	• New information centre on one site
	• Beacon site for roll out of the National Cancer Action Team (NCAT) Cancer Information Prescriptions programme

### Receiving a diagnosis

Receiving a diagnosis was an important touchpoint for all patients. Breast cancer patients remarked on a heightened sense of anxiety before their cancer was confirmed. Conversely, lung cancer patients spoke about not expecting a lung cancer diagnosis as many presented with ‘just a cough’. Both patient groups spoke of the importance of a diagnosis being communicated sensitively and a need for support immediately after diagnosis which is person-specific, delivered by a healthcare professional and allows time to process the information.

I was told I had cancer but nobody gave me any information. So it’s really weird when you go home and you say this to your husband, and he says, ‘Well, what does it mean?’ And he’s completely dependent on your slightly garbled account. They don’t give you too much information because they know you won’t take it in, but then of course straight afterwards you wish you did have all this information. (Breast04)

I would have really appreciated more time and personal contact with the clinical nurse specialist. It was left to the ward staff who were busy with other things. I felt it was a bit awkward. (Lung06)

This touchpoint became a co-design working group only in the lung service, where patients and staff worked together on implementing three specific improvements (Table 2).

### Being an inpatient

Inpatient experience was a touchpoint for both patient groups but breast cancer patients spoke less positively about their experiences—reporting a lack of psychological support, ‘feeling neglected’ and unfriendly staff. Breast cancer patients felt that specialist cancer wards would have offered staff greater knowledge of their disease and psychological needs.

What I did after a while was I didn’t really give myself morphine when I wanted it on the pump because I became too scared of what was going to happen if I wasn’t fully in control, and I don’t think that helped my recovery. (Breast09)

Mostly, they were admitted to general day surgery or surgical wards and, unlike lung cancer patients, treated in day surgery. Many described this as disorganised and chaotic, noting particular issues such as being separated prematurely from family and friends in the waiting area and feeling vulnerable in mixed-sex facilities. Lung cancer patients undergoing surgery were admitted to specialist cardiothoracic wards and largely reported more positive inpatient experiences.

Day surgery was identified as a priority for improvement at the breast service co-design event. Several improvement outcomes arising from this working group are shown in Table 1.

### Moving through the system

#### Continuity of care

The majority of patients reported feeling particularly vulnerable at certain points of the care pathway. Building relationships and establishing trust and confidence in healthcare professionals were particularly important for patients at these times. However, both patient groups spoke of a lack of continuity of care. Patients particularly highlighted the impact of having to retell their story to each new healthcare professional, in terms of both the process and the content of consultations. A lack of continuity eroded trust in the system as patients worried that things would be missed.

We saw four or five different doctors in oncology outpatients. What bothers me with that is that there’s the possibility to miss things whereas if there was continuity there, they would have a little bit more insight into what was actually going on. That worried me. (Lung04)

Continuity of care was identified as an improvement area at the lung cancer co-design event and several improvement outcomes from this working group were reported (Table 2).

#### Long waiting times stressful but justified

Long waiting times in outpatient clinics were a major touchpoint for both breast and lung cancer patients. However, patients felt long waiting times were justified if caused by other patients needing time with a healthcare professional.

…because of the stress levels…you can’t help but sit there worrying, especially if you’re waiting for results. And some people have to be dealt with longer than others; at one of my appointments, some person needed an hour of the consultants time, and I thought, ‘Oh, gosh, that could be me.’ So therefore you don’t moan and you don’t complain if you're sitting waiting. (Breast02)

Some patients suggested that this waiting time could be used more productively as an opportunity to offer more information:

Maybe that would have been a good opportunity for somebody to come and talk to the patients [about information/other services on offer]… because there is a lot of sitting around… a lot of wasted opportunities. (Lung01)

Issues related to outpatient clinics were identified at the breast co-design event as an improvement priority and Table 1 provides details of the changes made (some of which had a ‘knock on’ effect for the lung service—see Table 2).

#### Administrative processes

Both breast and lung cancer patients talked about the importance of efficient administrative processes for appointments and moving between services, and how they experienced feelings of uncertainty and disempowerment when appointments and letters were inaccurate or delayed.

I had to phone and phone and phone…its upsetting, frustrating. You get the feeling that you’re just a number, you’re just somebody on the end of a list. (Lung04)

A review of administrative processes was instigated as an improvement priority for the breast service and changes such as more efficient appointment processes were implemented (see Table 1).

### Understanding what is happening

Timely, clear, tailored information delivered personally by a healthcare professional (rather than via leaflets or books) was very important to all patients. Overall, both patient groups felt satisfied with the care and time spent with consultants and nursing staff.

…they spent hours, I mean literally hours, with us talking about the drug treatment that I was going to have, whether to have radiotherapy on both sides—I was really impressed with that. I never felt that they were under pressure, and they gave us all the information we could possibly want. (Breast05)

The surgeon explained everything to me. She went into detail, drew me pictures of what she was going to do…made sure I understood everything and I felt comfortable and reassured by this. (Lung07)

With the exception of diagnosis (see above), breast and lung patients were largely satisfied with the information received. However, both groups wanted more information at specific times in the pathway. Breast cancer patients expressed a need for more information about treatment side effects and what happens at the end of treatment. Lung cancer patients who had surgery expressed a need for more information about what happens after surgery.

I thought I would have had feedback after I had my surgery but I’ve had nothing. I’m waiting for the letters. No-one’s been in touch with me. That’s what they should give you before you go in for the operation—all the things that are going to happen to you. Do you have physiotherapy, why are you going to see a lung doctor, why are you going back to see the surgeon?… (Lung08)

The breast and lung co-design working groups both identified receiving information as a priority area for improvement. Improvements to the breast service are shown in Table 1 whilst improvements to lung service are shown in Table 2.

### Participant reflections on the key characteristics of the approach

Following the ‘review and celebration’ meeting (stage 6), four staff and five patients were interviewed about their involvement in the EBCD process. Interviewees highlighted four key characteristics of the EBCD approach: patient involvement, patient responsibility and empowerment, a sense of community, and a close connection between their experiences and the subsequent improvements that were made.

#### Patient involvement

In keeping with the philosophy underpinning the EBCD approach, participants confirmed that the high levels of direct patient involvement throughout the whole project had been a key feature of the work. Staff participants spoke of being ‘very moved by the fact that patients were being so honest … that was quite humbling’, ‘the constant feedback [being] really, really useful’, and ‘[needing] the eyeballing of each other to make it work’. Overall, participants commented on what was perceived as a significantly higher level of genuine direct patient and carer involvement (relative to other service improvement projects in which patients and staff had participated):

I don’t think as clinicians we can assume that we understand the patient experience of our service. I think we can feel that we may do, and we probably do. We have informal feedback from patients all the time about things that have gone well and things that maybe we can do better but if we want to really move forward and develop services that are truly patient centred, it is absolutely essential that we engage with patients and listen to their views about the services now and the service they want from us for the future.

#### Patient responsibility and empowerment

Related to the above characteristic, participants also spoke of how the EBCD process had given patients a greater sense of direct responsibility for the work and its outcomes (and as one staff member pointed out ‘they were doing our work for us!’):

One of the things that’s really good about patients being involved as well as staff is that you get to see both sides in a way that you can only do when you bring those two groups together. I think you could have a survey that you tick—I have done lots of surveys—I don’t think anything is happening necessarily with those surveys. I often feel that my experience isn’t reflected on there. I want to tick a box that’s not there. And this gave the patients a chance to say what was actually happening. (Breast09)

#### Sense of community

Key to the success of the project has been the strong relationship between patients and staff that has been built over time. This team or community aspect was remarked upon consistently by all those we interviewed:

…the patients are actually very supportive in the way in which they respond to the staff. That’s something that I found quite surprising about this as an approach. On paper, it might feel that it’s a bit confrontational, but the reality is that it’s very much about bringing about understanding between people who are coming at things from a different perspective.

#### The connection between experiences and improvements

The fourth and final characteristic is an important one, we believe, in terms of positioning EBCD in relation to other narrative-type approaches to improvement. It is the additional work that EBCD entails with regard to it also being an organisational development (OD) process (Bate and Robert 2007) that directly engages patients as well as staff in the design process from stories, through joint analysis and interpretation to implementation that distinguishes EBCD. Stories in themselves do not bring about change; it is the change process itself and the direct and active participation of staff and patients in it that produces implementation and action, and ultimately spread and sustainability. As one patient participant put it:

I was on the co-design group for communications and every meeting that we had, we would give our thoughts and we would be talking to our staff counterparts in a group and we would say, so and so wasn’t working and couldn’t we do this and couldn’t you do that?…putting forward suggestions…and the next meeting you would attend, it had already been implemented…so they were actually implementing things as we were going along, which was really positive. (Breast06)

The result of this ‘connection’ is, in our view, a much higher level of clinical engagement in the improvement effort than we have usually observed in other improvement projects, as suggested by the following quotation from another staff member:

And to have the doctors involved. I mean for the consultants to take time out to come and the senior nurses from outpatients to come in; it was a very big group and you know, wide group, so I thought it was very good.

## Discussion

Traditionally, efforts to understand the experiences of cancer patients have focused on specific tumour types. Both patient groups in this study identified similar touchpoints along their care pathways indicating that, fundamentally, cancer patients share the same concerns about their experiences of care. However, these generic touchpoints translated into improvement priorities specific to each tumour type. Many of the touchpoints emerging from the narrative patient interviews were modified through discussion at the staff/patient events to become priority areas for improvement. Hence, it appears that the co-design discussions in which patients and staff worked together to agree priorities were a crucial aspect of this EBCD intervention and its focus on patient-centred care[23]. More broadly, we would observe that all four of the characteristics highlighted above are about ‘engagement’ and ‘mobilisation’ which we argue manifested itself in particularly high levels of clinical involvement, support and enthusiasm for the work. Having watched the patient films, heard the patient stories and discussed their experiences with the patients they themselves had treated and cared for, clinical staff felt it to be an imperative and a responsibility to do something about improving ‘their’ service and those experiences; otherwise, they could not live comfortably with themselves as professional carers.

Of course, there are limitations to the approach. Quality improvement interventions always need to be tailored to specific services (i.e. adapted to a particular illness trajectory, service structure, available resources—including staff time—and work practices etc.) and their success will be, at least, partly dependent on the receptiveness of the local context[24–26]. Additionally, EBCD does not explicitly seek to recruit a representative sample of patients but rather, seeks informants who can ‘tell their story’ and potentially participate in the co-design work. This can lead to the needs of ‘harder to reach’ patient groups remaining unheard. Nonetheless, a co-design approach to service improvement at least offers the opportunity for motivated patients to reflect collectively and collaboratively with the staff directly providing their care and the translation of touchpoints into improvement priorities in the project presented here illustrates how specific priorities may vary in significant ways between tumour groups.

These findings suggest that cancer patients may have generic concerns[27] (at a high level of abstraction) but co-design improvements need to be service specific—being more targeted and effective when they arise from shared priority setting between patients and staff. The co-design process led to an increased understanding of the ‘other’ perspective, resulting in a potentially broader cultural change in mindsets and behaviour. Certainly staff participating in the co-design groups reported a greater sense of empowerment to make changes to their service. It is important to determine whether these changes are sustainable over time but it should be noted that some co-design working groups disbanded early. These often comprised multidisciplinary staff who had not previously worked together, suggesting the importance of establishing or facilitating teamworking as an integral part of the approach. The indirect benefits of the implementation of the EBCD approach in this centre are being evaluated in an ongoing spread and sustainability study.

The configuration of cancer services leads to a focus on the differences between tumour-specific groups. Improving outcomes guidance[28] and the process of peer review encourage reflection within, rather than across, services[29]. Our findings suggest that breast and lung cancer patients identified generic touchpoints that translated into service-specific improvement priorities. Healthcare leaders and cancer-care practitioners should focus on generic touchpoints in order to then identify specific problems within local services, ensuring that the importance of the patient voice and close collaboration with staff is present throughout any improvement project.

Given the increasingly influential notion of co-designing public services (‘service development driven by the equally respected voices of users, providers and professionals’)[30, 31] and cancer services specifically,[28, 32] EBCD represents one approach to reposition (largely) passive recipients of a service as more active consumers and citizens in a coalition, or partnership,[33] with staff. The approach described here seeks to equip users and providers to work together on service and quality improvement offering patients and carers a much stronger voice in initiatives that explicitly strive to improve their experiences.
